# Nitrofurantoin Toxicity: A Near Case of Mistaken Identity

**DOI:** 10.7759/cureus.3315

**Published:** 2018-09-17

**Authors:** Jeffrey A Miskoff, Moiuz Chaudhri

**Affiliations:** 1 Internal Medicine, Jersey Shore University Medical Center, Neptune City, USA

**Keywords:** nitrofurantoin, mistaken, toxicity, small cell lung cancer, hyperplasia

## Abstract

Nitrofurantoin is one of the most utilized antibiotics to treat bladder and urinary tract infections (UTIs). Despite the clinical benefits, it requires vigilant monitoring, as it can cause damage to multiple organs, especially the lungs and the liver. This case is an example of clinical vigilance, which provided tremendous benefit for the patient.

## Introduction

Nitrofurantoin is an antibacterial agent commonly used in the management of urinary tract infections (UTIs). It is frequently used in the management of acute cystitis and patients with recurrent bladder infections. Nitrofurantoin may cause problems involving different organ systems after short-term or chronic use. Liver and lung toxicity are among the most common organs affected. A majority of the patients presenting with pulmonary manifestations are women ranging in age from 60 to 70 years [1]. A recent study suggested that people 65 or older have a significantly higher rate of suffering from toxicity due to the chronic use of nitrofurantoin (adjusted relative risk of 1.53 with 95% CI of 1.04-2.24). Acute toxicity presents with changes at the molecular level of the lungs along with a different mechanism [2].

## Case presentation

**Figure 1:**
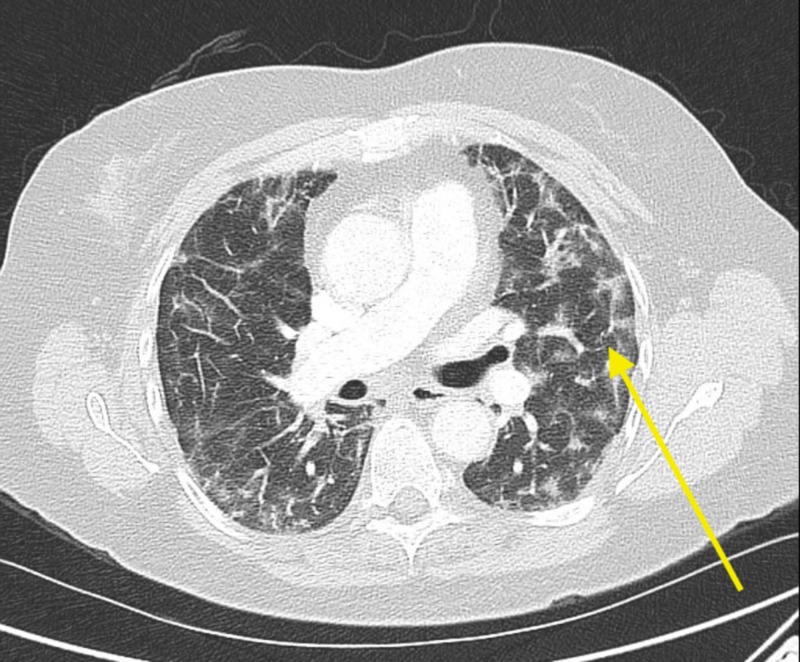
A computed tomography (CT) scan showing opacities described as pneumonia along with patchy infiltrates (arrow).

Here, we present a case of a 60-year-old established patient of ours who was initially seen on January 12, 2013, for symptoms related to a paraesophageal hernia. The patient started nitrofurantoin 100 mg daily on October 26, 2013, and it was discontinued on May 1, 2014. Later in the year, the patient developed a productive cough, which did not respond to the outpatient antibiotics. In 2014, the patient presented with a dry cough, a chest x-ray depicting patchy bilateral infiltrates, and a subsequent computed tomography (CT) scan illustrating bilateral opacities described as pneumonia (Figure 1). The patient did not respond to a brief trial of outpatient oral steroidsand antibiotics. A lack of improvement in symptomology led to bronchoscopy, which was unremarkable. Additionally, no densities or masses were identified during the procedure. The patient was also worked up for aspiration by bronchoalveolar lavage (BAL) with cytology brushing and multiple transbronchial biopsies of the right upper lung. Aspiration pneumonia was suspected. Prior to the procedure and the discontinuation of nitrofurantoin, the patients’ aspartate aminotransferase (AST) test was 222 units/liter (reference range 10-42 units/liter) and alanine aminotransferase (ALT) test was 153 units/liter (reference range 10-60 units/liter) along with a low albumin of 3.4 g/dl (reference range 3.5-5.0 g/dl). After the discontinuation of the antibiotic, the patients AST and ALT decreased to 112 and 123 units/liter, respectively.

**Figure 2:**
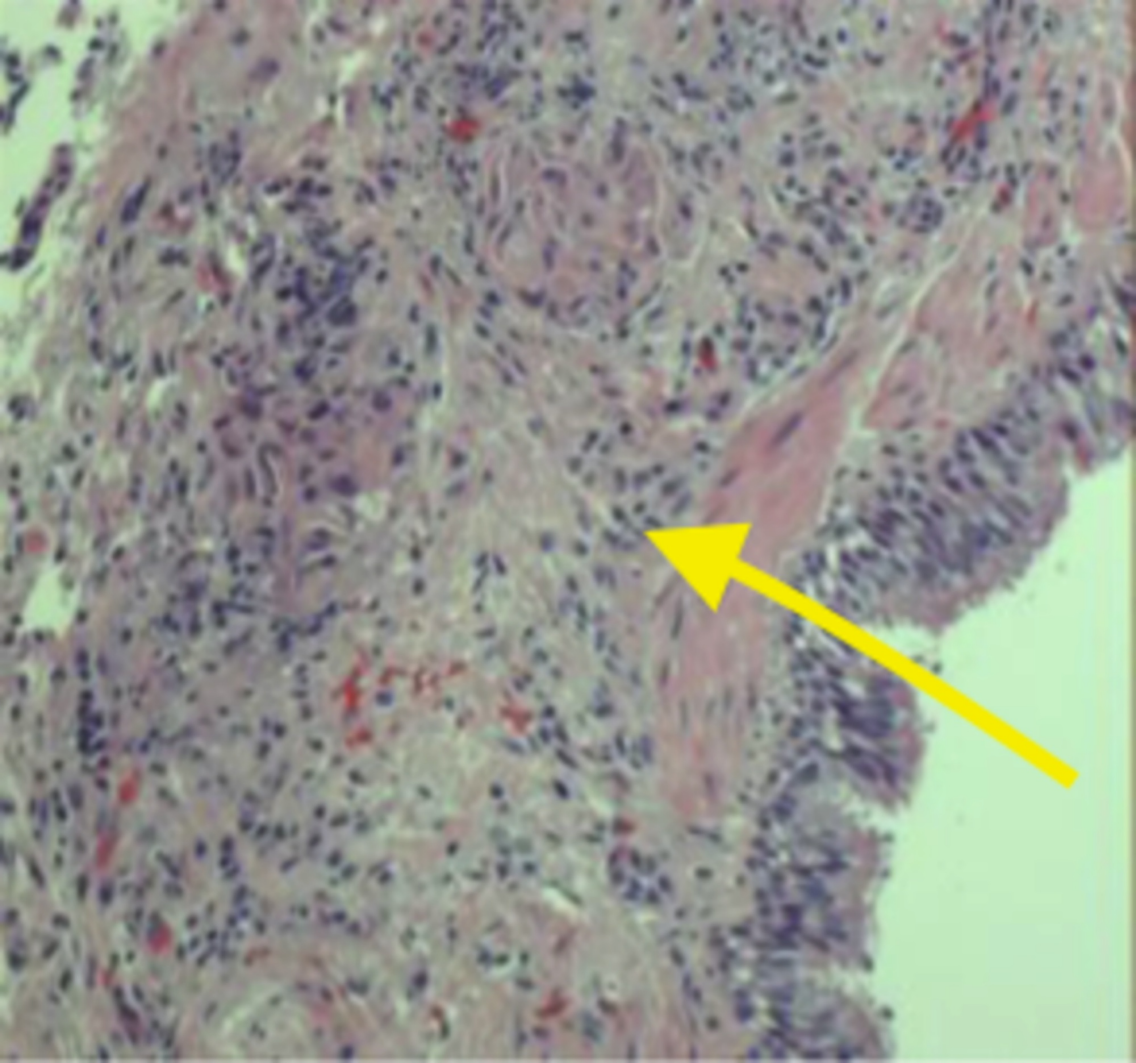
Epithelial macrophages along with atypical pneumonic cystic hyperplasia suggesting a neoplasm (arrow).

Although workup did not reveal the typical presentation of a malignancy or a lung mass, nitrofurantoin was held as a precautionary measure especially since the patient has been using it chronically. A laboratory investigation of the bronchial aspirate suggested moderate growth of normal oropharyngeal flora and scant Candida albicans, the gram stain revealed gram-positive cocci in pairs and rare epithelial cells, and no acid-fast bacilli were recovered. Additionally, lavage was negative for malignant cells but epithelial, squamous and pulmonary macrophages were present. A surgical pathology analysis exhibited atypical pneumonic cystic hyperplasia with a cluster of cells suspicious for neoplasm on initial evaluation by the head of pathology (Figure 2). A preliminary phone call to the pulmonologist was made because of the suspicion of a primary small cell lung carcinoma (SCLC). The pathologist inquired more about the case and more specifically if there was a lung mass on imaging. After further discussion, a final path report excluded carcinoma from the diagnosis even though the initial review of the samples showed cells resembling oat cells.

**Figure 3:**
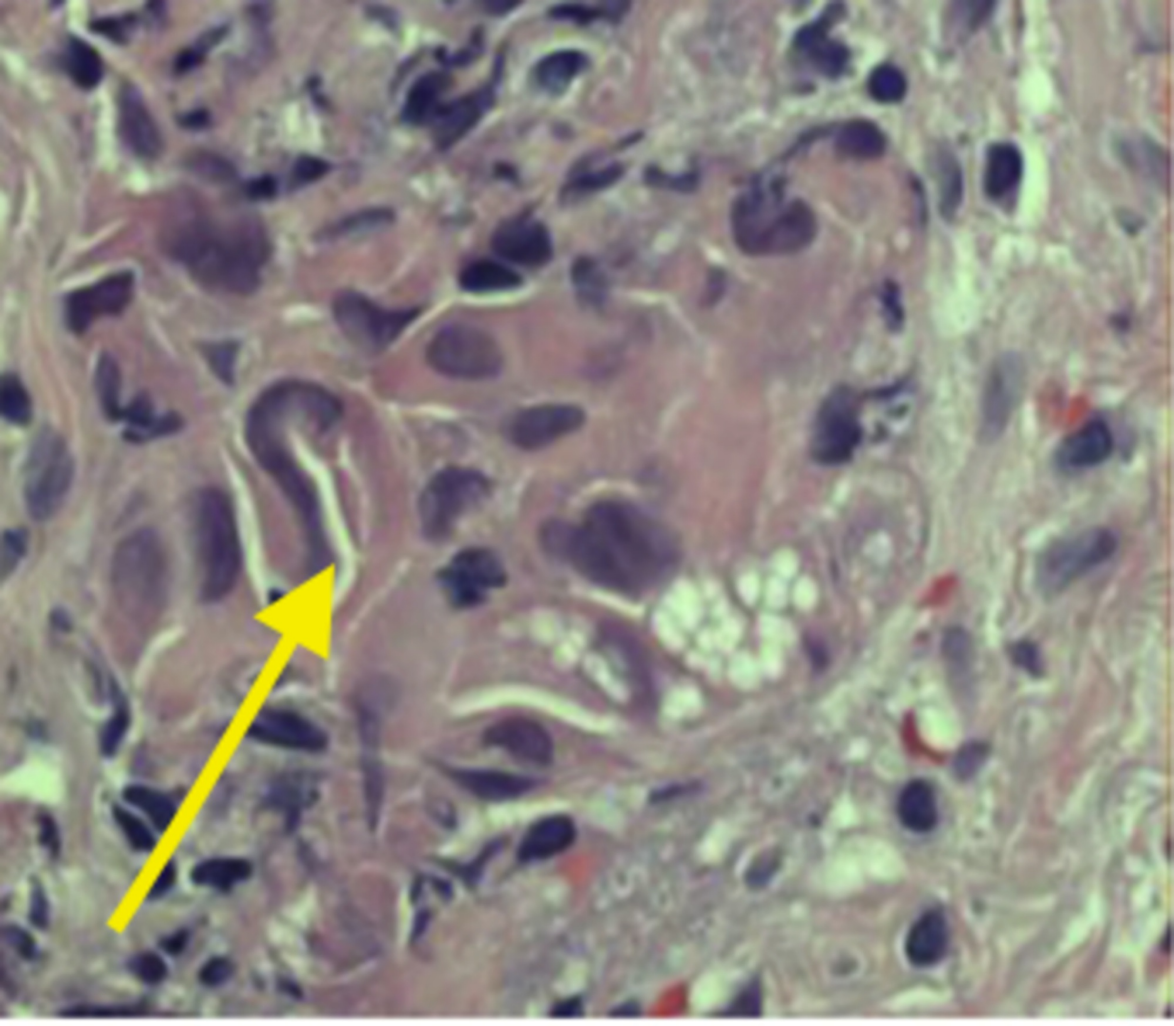
Alveoli surrounded by enlarged atypical pneumocytes with a powdery chromatic pattern and small nuclei (arrow).

**Figure 4:**
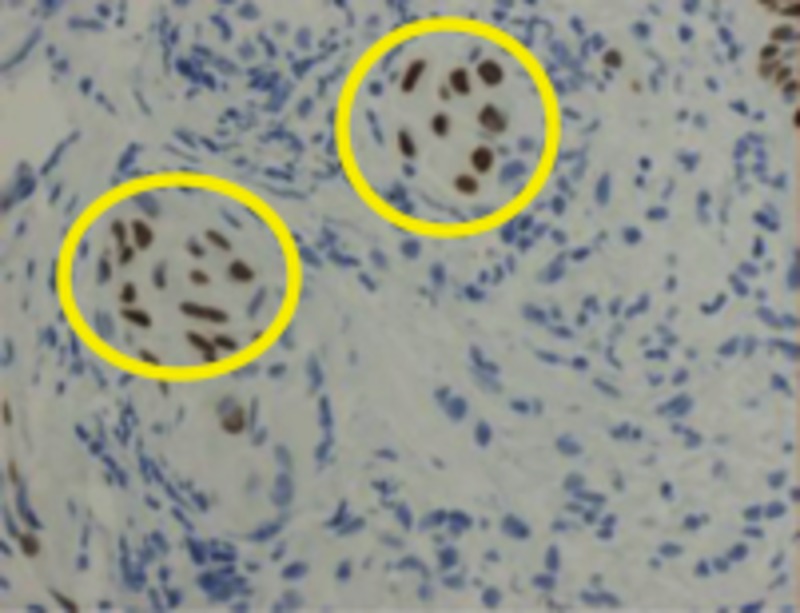
Cluster of cells within the submucosa isolated from the alveolar epithelium (circles).

A peribronchial tissue analysis under the guidance of microscopy showed pseudostratified ciliated columnar epithelium (normal finding), alveoli were lined by enlarged, atypical appearing pneumocytes with a powdery chromatic pattern and small nuclei (Figure 3). The interstitium appeared thickened, with fibrotic and chronic inflammatory cells noted. Also, adjacent peribronchial tissue contained a chronic inflammatory infiltrate, which was mild, and presented with two nests of very atypical cells. The two clusters of cells were within the submucosa without a relationship with the alveolar epithelium (Figure 4). Lastly, the nuclei of the adjacent peribronchial presented with irregularly shaped nuclei, with an irregular distribution of the chromatin pattern and prominent nucleoli. To investigate this step further, two clusters of cells were positive for cytokeratin 7 (CK 7d) and thyroid transcription factor-1 (TTF). However, the sample was negative for cytokeratin 20 (CK20) and a tumor protein 40 (P40). Molecular evidence suggests that the presence of CK20 and CK7 is strongly associated with advanced malignancy and TTF-1 is found in type II pneumocytes and Clara cells, suggesting a better survival rate [3-4]. Lastly, P40 is known to be a highly specific marker of squamous cell carcinoma.

## Discussion

Nitrofurantoin is frequently used to treat bladder infections along with providing prophylactic coverage to the patient. Nitrofurantoin can lead to pulmonary conditions presenting in an acute and chronic manner [5-6]. An acute pulmonary reaction presents with interstitial inflammation, focal hemorrhage, and reactive type II pneumocytes. Evidence indicates that the acute onset of symptoms involves hypersensitivity reaction type I or III; the mean onset of hypersensitivity pneumonitis originating from nitrofurantoin is usually seen after 8.7 days [7]. Repeat exposure significantly decreases the time needed for acute symptoms to present [6,8].

Chronic reactions can present with significant interstitial pneumonitis, a thickening of the alveolar septa, and, infrequently, vascular sclerosis [9-10]. Less common presentations of chronic reactions can present with chronic eosinophilic pneumonia and desquamative interstitial pneumonia with an abundance of macrophages in the alveoli. The chronic onset of toxicity can be due to cell-mediated or toxin insult [11-12]. Evidence points toward nitrofurantoin metabolites being the culprit by damaging lung microsomes. In addition, it can cause hepatotoxicity, a granulomatous reaction, and autoimmune-mediated hepatitis, which can lead to death [13]. Clinical evidence suggests that nitrofurantoin can lead to sensorimotor polyneuropathy along with other neurotoxic events [14].

Nitrofurantoin is one of many medications that can have negative effects on patients. This case is unique in the fact that the preliminary read from the pathologist was concerning for SCLC. Fortunately, the patient did not have a lung mass or other clinical findings to support this diagnosis. Further review of the pathology and the case ultimately led to the correct diagnosis of lung toxicity from chronic nitrofurantoin use. After approximately four to six weeks of 0.5 mg/kg prednisone, the patient clinically improved. Although the patient was lost to follow-up, a recent phone conversation with her confirmed that she does not have any residual pulmonary symptoms or findings to report.

## Conclusions

Pulmonary drug toxicity may present with different imaging patterns and atypical cells on pathology. Although uncommon, nitrofurantoin, especially when used chronically, may present challenging diagnostic and treatment dilemmas. A careful review of pathology, imaging, and clinical history and presentation were key in order to make the correct diagnosis in this case. 

## References

[1] Mendez J, Nadrous H, Hartman T, Ryu J (2005). Chronic nitrofurantoin-induced lung disease. Mayo Clin Proc.

[2] Santos J, Batech M, Pelter M, Deamer R (2016). Evaluation of the risk of nitrofurantoin lung injury and its efficacy in diminished kidney function in older adults in a large integrated healthcare system: a matched cohort study. J Am Geriatr Soc.

[3] Hernandez B, Frierson H, Moskaluk C (2005). CK20 and CK7 protein expression in colorectal cancer: demonstration of the utility of a population-based tissue microarray. Hum Pathol.

[4] Misch D, Blum T, Boch C (2015). Value of thyroid transcription factor (TTF)-1 for diagnosis and prognosis of patients with locally advanced or metastatic small cell lung cancer. Diagn Pathol.

[5] Huttner A, Verhaegh E, Harbarth S, Muller A, Theuretzbacher U, Mouton J (2015). Nitrofurantoin revisited: a systematic review and meta-analysis of controlled trials. J Antimicrob Chemother.

[6] Kabbara W, Kordahi M (2015). Nitrofurantoin-induced pulmonary toxicity: a case report and review of the literature. J Infect Public Health.

[7] Sovijarvi A, Lemola M, Stenius B, Idanpaan-Heikkila J (1977). Nitrofurantoin-induced acute, subacute and chronic pulmonary reactions. Scand J Respir Dis.

[8] Hainer B, White A (1981). Nitrofurantoin pulmonary toxicity. J Fam Pract.

[9] Fenton M, Kanthan R, Cockcroft D (2008). Nitrofurantoin-associated bronchiolitis obliterans organizing pneumonia: report of a case. Can Respir J.

[10] Kanji Z, Su V, Mainra R (2011). Nitrofurantoin-induced pulmonary reaction involving respiratory symptoms: case report. Can J Hosp Pharm.

[11] Bhullar S, Lele S, Kraman S (2007). Severe nitrofurantoin lung disease resolving without the use of steroids. J Postgrad Med.

[12] Cameron R, Kolbe J, Wilsher M, Lambie N (2000). Bronchiolitis obliterans organising pneumonia associated with the use of nitrofurantoin. Thorax.

[13] Sakaan S, Twilla J, Usery J, Winton J, Self T (2014). Nitrofurantoin-induced hepatotoxicity: a rare yet serious complication. South Med J.

[14] Grill M, Maganti R (2011). Neurotoxic effects associated with antibiotic use: management considerations. Br J Clin Pharmacol.

